# Supplementary material for: Active Food Packaging Coatings Based on Hybrid Electrospun Gliadin Nanofibers Containing Ferulic Acid/Hydroxypropyl-Beta-Cyclodextrin Inclusion Complexes
Source: Nanomaterials (Basel). 2018 Nov 7;8(11):919. doi: 10.3390/nano8110919 (PMC6266051; doi:10.3390/nano8110919)
Supplement: Supplementary file 1 [file nanomaterials-08-00919-s001.pdf]

Supplementary Materials

Active Food Packaging Coatings Based on Hybrid Electrospun Gliadin Nanofibers Containing Ferulic Acid/Hydroxypropyl-Beta-Cyclodextrin Inclusion Complexes

Niloufar Sharif [1], Mohammad-Taghi Golmakani [1], Mehrdad Niakousari [1], Seyed Mohammad Hashem Hosseini [1], Behrouz Ghorani [2], Amparo Lopez-Rubio [3,*]

[1] Department of Food Science and Technology, School of Agriculture, Shiraz University, km 12 Shiraz-Esfahan Highway, 71441-65186 Shiraz, Iran; sharif1986@shirazu.ac.ir (N.S.); golmakani@shirazu.ac.ir (M.-T.G.); niakosar@shirazu.ac.ir (M.N.); hhosseini@shirazu.ac.ir (S.M.H.H.)

[2] Department of Food Nanotechnology, Research Institute of Food Science and Technology (RIFST), km 12 Mashhad-Quchan Highway, 91895/157/356 Mashhad, Iran; b.ghorani@rifst.ac.ir

[3] Food Quality and Preservation Department, IATA-CSIC, 46980 Paterna, Valencia, Spain

* Correspondencce: amparo.lopez@iata.csic.es; Tel.: +34-963-900-022; Fax: +34-963-636-301

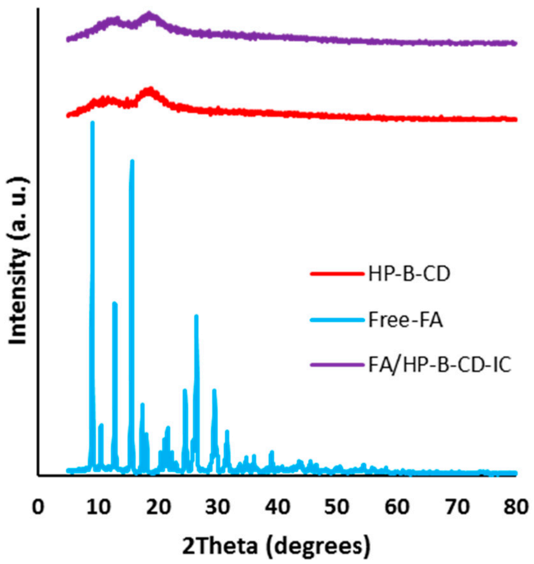

Figure S1. X-ray diffraction (XRD) diffraction patterns of pure FA, HP-β-CD and FA/HP-β-CD-IC.

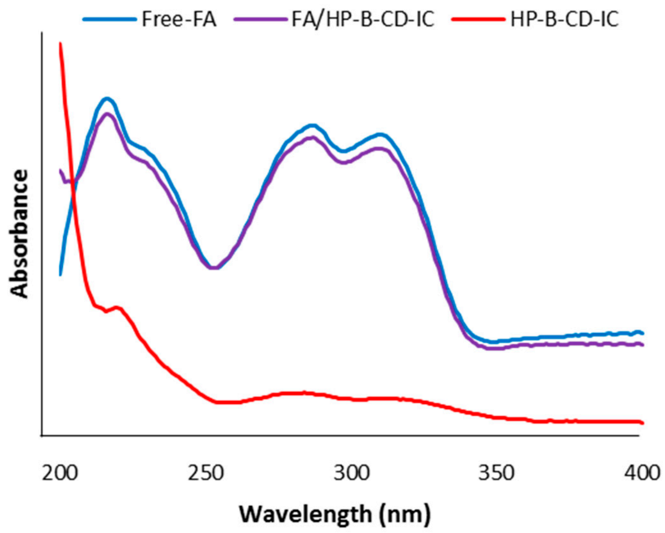

Figure S2. UV-visible absorption spectra of pure FA, HP-β-CD and FA/HP-β-CD-IC.

Table S1. Temperatures of maximum degradation rate and corresponding weight losses of the different degradation stages and residual matter at 700°C from the various samples.

Sample	T_{max1} (°C) [a]	WL_1 (%) [b]	T_{max2} (°C) [a]	WL_2 (%) [b]	T_{max3} (°C) [a]	WL_3 (%) [b]	RM (%) [c]
FA	-	-	252.6	43.8	442.2	81.4	3.9
G-FA	67.7	1.4	175.8	8.6	312.6	35.2	20.3
FA/HP-B-CD-IC	80.9	7.1	-	-	350.4		8.9
G-FA/HP-B-CD-IC	74.9	7.2	-	-	317.9	40.8	11.7
G	79.9	7.3	-	-	322.2	37.3	21.6

[a] Temperature of maximum degradation rate. [b] Weight loss of the corresponding degradation stage.

[c] Residual matter (RM) at 700 °C.
